# GP73 promotes invasion and metastasis of bladder cancer by regulating the epithelial–mesenchymal transition through the TGF‐β1/Smad2 signalling pathway

**DOI:** 10.1111/jcmm.13442

**Published:** 2018-01-19

**Authors:** Han‐Jie Yang, Ge‐Liang Liu, Bo Liu, Tian Liu

**Affiliations:** ^1^ Department of Urology Pingxiang Affiliated, Southern Medical University Pingxiang China; ^2^ Department of General Surgery Xiangya 2nd Hospital of Central South University Changsha China

**Keywords:** bladder cancer, Golgi membrane protein 73, TGF‐β1/Smad2 signalling pathway, epithelial–mesenchymal transition, invasion, metastasis

## Abstract

This study investigated the effects of Golgi membrane protein 73 (GP73) on the epithelial–mesenchymal transition (EMT) and on bladder cancer cell invasion and metastasis through the TGF‐β1/Smad2 signalling pathway. Paired bladder cancer and adjacent tissue samples (102) and normal bladder tissue samples (106) were obtained. Bladder cancer cell lines (T24, 5637, RT4, 253J and J82) were selected and assigned to blank, negative control (NC), TGF‐β, thrombospondin‐1 (TSP‐1), TGF‐β1+ TSP‐1, GP73‐siRNA‐1, GP73‐siRNA‐2, GP73‐siRNA‐1+ TSP‐1, GP73‐siRNA‐1+ pcDNA‐GP73, WT1‐siRNA and WT1‐siRNA + GP73‐siRNA‐1 groups. Expressions of GP73, TGF‐β1, Smad2, p‐Smad2, E‐cadherin and vimentin were detected using RT‐qPCR and Western blotting. Cell proliferation, migration and invasion were determined using MTT assay, scratch testing and Transwell assay, respectively. Compared with the blank and NC groups, levels of GP73, TGF‐β1, Smad2, p‐Smad2, N‐cadherin and vimentin decreased, and levels of WT1 and E‐cadherin increased in the GP73‐siRNA‐1 and GP73‐siRNA‐2 groups, while the opposite results were observed in the WT1 siRNA, TGF‐β, TSP‐1 and TGF‐β + TSP‐1 groups. Cell proliferation, migration and invasion notably decreased in the GP73‐siRNA‐1 and GP73‐siRNA‐2 groups in comparison with the blank and NC groups, while in the WT1 siRNA, TGF‐β, TSP‐1 and TGF‐β + TSP‐1 groups, cell migration, invasion and proliferation showed the reduction after the EMT. These results suggest that GP73 promotes bladder cancer invasion and metastasis by inducing the EMT through down‐regulating WT1 levels and activating the TGF‐β1/Smad2 signalling pathway.

## Introduction

Bladder cancer is the ninth most frequent cancer in the world, with approximately 430,000 new cases diagnosed every year 1. The occurrence and progression of bladder cancer are a complex process involving multiple factors and steps that are influenced by molecular genetics and environmental and chemical factors 1, 2, 3. Recent studies have shown that the occurrence and progression of bladder cancer are closely related to alterations to certain signalling pathways, such as abnormalities in the expression patterns of epidermal growth factors, including domain‐7 (EGFL7), E‐cadherin proteins, and in the PI3K‐Akt‐mTOR signalling pathway 4, 5, 6. Among the altered pathways, the transforming growth factor (TGF)‐β1 signal pathway has attracted much attention 7.

TGF‐β is an important cytokine that plays an important role in cell proliferation, differentiation, angiogenesis and embryonic development 8. TGF‐β1 is one of the three isomers of TGF‐β (TGF‐β1, TGF‐β2 and TGF‐β3) 9, 10. TGF‐β inhibits the proliferation of tumour cells in early‐stage tumours and promotes cell apoptosis, and it also functions as a tumour promoter for tumour invasion and metastasis during oncogenesis 11. It was reported that the TGF‐β1‐induced epithelial–mesenchymal transition (EMT) is mediated by the transcriptional activation of Sonic hedgehog (Shh) in non‐small‐cell lung cancer 12. The EMT refers to the transformation of polar epithelial cells into free‐moving interstitial cells, thereby enabling the migration and invasion of tumour cells 13. Smad is a signal transduction protein that consists of eight proteins (Smad1‐8) that mediates signal transduction in the TGF‐β1 pathway 14, 15. Smad2 was found to be activated by TGF‐β1 *via* TGF receptors on epithelial cells 16. The TGF‐β‐induced Smad signalling pathway has been extensively studied with the aim of understanding the complex and versatile responses governing tumour metastasis, increased motility, invasiveness and the EMT 17. A recent study has demonstrated that Golgi membrane protein 73 (GP73) is highly expressed in tumour cells and acts as a potential cancer cell marker 18. Moreover, GP73 was reported to be correlated to EMT‐related molecules in hepatocellular carcinoma (HCC) 19. In addition, previous research has shown that Golgi phosphoprotein 2 (GOLPH2, also termed GOLM1 and GP73) deletion results in increased Wilms' tumour gene (WT1) expression 20. Nevertheless, the activity of the GP73/TGF‐β1/Smad2 pathway in the regulation of the EMT in bladder cancer has not been studied. Thrombospondin‐1 (TSP‐1) contains three type I repeats, and TSP‐1 3TSR (all three TSRs of the type 1 repeat domain) can activate TGF‐β1 21. TSP‐1, as a TGF‐β signalling activator, has been reported to regulate the activation of the TGF‐β signal pathway during liver regeneration 22. In this study, we aimed to elucidate the role of GP73 in regulating the EMT to promote the invasion and metastasis of bladder cancer through the TGF‐β1/Smad2 signalling pathway and to establish a theoretical foundation for the discovery of new molecular targets in the clinical treatment of bladder cancer.

## Materials and methods

### Ethical statement

This study was performed in accordance with the guidelines established by the Medicine Ethics Review Committee of Pingxiang Affiliated Hospital, Southern Medical University, and all patients signed a consent form.

### Study subjects

From March 2012 to March 2014, a total of 102 patients with bladder cancer were selected from Pingxiang Affiliated Hospital, Southern Medical University. Bladder cancer and adjacent tissue samples (bladder epithelial mucosa tissues at a distance of over 5 cm from the edge of cancer tissues) were obtained. All specimens were confirmed by pathological examinations. The patients included 54 males and 48 females with a mean age of 67 years (range: 51–85 years). Pathological grades of the tissue specimens were assessed in accordance with the World Health Organization/International Society of Urological Pathology (WHO/ISUP) 2004 edition of bladder cancer standards 23 and the 2002 Union for International Cancer Control (UICC) standards for tumour node metastasis (TNM) staging and pathological diagnosis 24. There were 17 cases of low malignant potential (LMP) papillary urothelial cancer, 35 cases of low grade (LG) urothelial carcinoma and 50 cases of high grade (HG) urothelial carcinoma. There were 52 cases of non‐muscle‐invasive bladder cancer (NMIBC) and 50 cases of muscle‐invasive bladder cancer (MIBC). There were 65 cases at stage I‐II and 37 cases at stage III‐IV. Additionally, there were 35 cases with lymph node metastases (LNMs) and 67 cases without LNMs. None of the patients received chemotherapy, radiotherapy or biological therapy prior to tissue collection. Normal bladder tissue samples (bladder epithelial mucosa tissues) for comparison with the bladder cancer group were obtained from 106 individuals who had undergone surgery for reasons other than bladder cancer and who had no significant disease. These samples were collected from 79 males and 27 females with a mean age of 62 years (range: 46–82 years). All specimens were fixed in 10% formalin and embedded in paraffin for subsequent experiments.

### Immunohistochemistry (IHC)

The paraffin‐embedded specimens were cut into 4‐μm serial sections. The sections were dried at 60°C for 1 hr, deparaffinized using a conventional xylene method and then dehydrated in a graded alcohol series. They were then incubated in 3% H_2_O_2_ (Sigma‐Aldrich Chemical Company, St Louis, MO, USA) at 37°C for 30 min., washed with phosphate‐buffered saline (PBS), boiled in 0.01 M citrate buffer at 95°C for 20 min., cooled to room temperature and washed with PBS. The samples were then blocked with normal sheep serum at 37°C for 10 min. and incubated overnight with primary antibodies for GP73 (ab109628), TGF‐β1 (ab64715), E‐cadherin (ab197751), Smad2 (ab33875) and vimentin (ab8978) (1:400 dilutions; Abcam Inc., Cambridge, MA, USA) at 4°C and then washed with PBS. The samples were then incubated with horseradish peroxidase‐labelled secondary antibodies (Bioss, Beijing, China) for 30 min. and treated with diaminobenzidine (Sigma‐Aldrich Chemical Company). Finally, the samples were stained with haematoxylin (Bogoo, Shanghai, China) and mounted. PBS was used as the negative control (NC) instead of a primary antibody, and positive sections were used as positive controls. In each slide‐mounted sample, five fields of view were randomly selected at high magnification (×400), with each field containing 100 cells. The samples were scored according to the percentage of positive cells: samples were considered positive (+) if positive tumour cells/all tumour cells were >10% and negative (−) if positive cells were ≤10% 25. Scoring of the IHC assay was performed in the double‐blinded method by two operators.

### Cell culture

The cell lines used in this study included the human bladder cancer T24 and RT4 cell lines (ATCC, Manassas, VA, USA), the 253J and J82 cell lines (both provided by professor Leland WK Chung from Emory University in the United States), and the 5637 cell line (Chinese Academy of Science shanghai branch, Shanghai, China). The T24 and RT4 cell lines were cultured in McCoy's 5A medium (Gibco, Gaithersburg, MD, USA) containing 10% foetal bovine serum (FBS) (HyClone, Logan, UT, USA), while the 253J, J82 and 5637 cell lines were cultured in Dulbecco's modified Eagle's medium (DMEM) (GIBCO BRL, Eggenstein, Germany) containing 10% FBS. All cell lines were cultured in a 5% CO_2_ incubator at 37°C. The cells were digested with 0.25% trypsin (Gibco). T24 and RT4 cells were dissociated into single‐cell suspensions using McCoy's 5A medium containing 10% FBS, and the 253J, J82 and 5637 cells were dissociated into single‐cell suspensions using DMEM containing 10% FBS. The cells were passaged using standard procedures, and cells in the logarithmic growth phase were used in the experiments.

### Screening siRNA against WT1

Twenty‐four hours after transfection, cells in the logarithmic growth phase were seeded in a six‐well plate. Once reaching 30% ~ 50% confluency, cells were subjected to transfection. According to the ExPASy, the half‐life of WT1 protein was 30 hrs. Three different WTI siRNA sequences (Table 1) were designed. Diluted WTI siRNA‐1, ‐2, ‐3 and NC siRNA were separately incubated in the Lipofectamine 2000TM RNAiMAX (Lip2000; Invitrogen Inc., Carlsbad, CA, USA) at room temperature for 20 min. to form the corresponding siRNA‐Lip2000 products. The cells were maintained in the RPMI 1640 culture medium supplemented with the corresponding siRNA‐Lip2000 products for 24 and 48 hrs (Table 1).

**Table 1:** WT1 siRNA sequence

	Forward	Reverse
WT1 siRNA‐1	UAUAAGUACUAGAUGCAUCAC	GUGAUGCAUCUAGUACUUAUA
WT1 siRNA‐2	AUGAACUUAGGAGCCACCUU	AAGGUGGCUCCUAAGUUCAUC
WT1 siRNA‐3	GCAGCUAACAAUGUCUGGUUA	UAACCAGACAUUGUUAGCUGC
NC siRNA	UUCUCCGAACGUGUCACAGUTT	ACGUGACACGUUCGGAGAATT

WT1, Wilms' tumour gene 1; NC, negative control.

### Cell transfection and grouping

253J and 5637 cells in the logarithmic growth phase were seeded into six‐well plates. Upon reaching 30% ~ 50% confluency, cells were transfected using Lipofectamine 2000 (Invitrogen Inc.) according to the manufacturer's instructions. GP73‐siRNA (100 pmol) and the NC sequence (50 nM) were diluted using 250 μl of Opti‐MEM serum‐free medium (Gibco) and incubated at room temperature for 5 min. Similarly, Lipofectamine 2000 (5 μl) was diluted with 250 μl of Opti‐MEM serum‐free medium and then incubated at room temperature for 5 min. The above two solutions were then mixed, incubated at room temperature for 20 min. and then added to the cell culture plates. After incubating at 37°C with 5% CO_2_ for 6 ~ 8 hrs, the cells were cultured in complete medium for an additional 48 hrs. The cells were classified into the blank, NC (transfected with an NC siRNA), TGF‐β(treated with 5 ng/ml of TGF‐β) 26, TGF‐β + thrombospondin‐1 (TSP‐1) (treated with 5 ng/ml TGF‐β for 8 hrs before the treatment with 5 ng/ml TSP‐1) 25, TSP‐1 (containing 5 ng/ml of TSP‐1), GP73‐siRNA‐1 (transfected with GP73‐siRNA‐1), GP73‐siRNA‐2 (transfected with GP73‐siRNA‐2), WT1‐siRNA (transfected with WT1‐siRNA), WT1‐siRNA + GP73‐siRNA‐1 (GP73‐siRNA‐1+ transfected with WT1‐siRNA) a TSP‐1+ GP73‐siRNA‐1 (transfected with GP73‐siRNA‐1 for 8 hrs before the treatment with 5 ng/ml TSP‐1) and GP73‐siRNA‐1+ pcDNA‐GP73 groups (transfected with GP73‐siRNA‐1 for 8 hrs before the transfection of pcDNA‐GP73: GP73 without the 3′‐UTR was generated by PCR amplification and then cloned into the mammalian expression vector pcDNA 3.1) (Table 2) 27. All siRNA sequences were designed and synthesized by GenePharma (Shanghai, China), and the pcDNA 3.1 was from Invitrogen Inc.

**Table 2:** GP73‐siRNA and NC sequences

	Forward	Revers
GP73 siRNA‐1	CUGCCUGACAUAUUUGGGCATT	UGUUCAAAUUCUAAGCCACTT
GP73 siRNA‐2	GAACAUAGUUCUCCUUCAATT	UUGAAGGAGAACUAUGUUCTT
NC siRNA	UUCUCCGAACGUGUCACAGUTT	ACGUGACACGUUCGGAGAATT

GP73, Golgi membrane protein 73; NC, negative control.

### Reverse transcription quantitative polymerase chain reaction (RT‐qPCR)

T24, 5637, RT4, 2537J and J82 cells in the logarithmic growth phase were collected. The contents of GP73, E‐cadherin, N‐cadherin and vimentin in those cells were determined using RT‐qPCR to screen the cell lines. The mRNA levels of GP73, TGF‐β1, Smad2, E‐cadherin, N‐cadherin, vimentin and WT1 were determined in the transfected cells.

Total RNA was extracted using a miRNeasy Mini Kit (Qiagen, Valencia, CA, USA). The absorption values at 260 and 280 nm, which were measured using an ultraviolet spectrophotometer, were used to calculate the concentration and purity of the extracted RNA. A 260/280 optical density (OD) ratio of 1.7–2.1 indicated that the purity level was high enough to permit further experiments. A PCR Amplification kit (Applied Biosystems, Foster City, CA, USA) was used to reversely transcribe sequences to a cDNA template. An ABI7500 quantitative PCR instrument (Applied Biosystems,) was used for the RT‐qPCR analysis. The reaction conditions were as follows: pre‐denaturation at 95°C for 10 min., denaturation at 95°C for 10 sec., annealing at 60°C for 20 sec. and extension at 72°C for 34 sec., which ran for 40 cycles. The reaction system contained 10 μl of SYBR Premix Ex Taq TM II, 0.8 μl of PCR forward primer (10 μM), 0.8 μl of PCR reverse primer (10 μM), 0.4 μl of ROX reference dye, 2.0 μl of cDNA template and 6.0 μl of sterilized distilled water. Table 3 shows the primer sequences used in this study. The glyceraldehyde‐3‐phosphate dehydrogenase (GAPDH) gene served as the internal control. Comparisons of gene expression between experimental and control groups were performed using the 2^−ΔCt^ method: ΔCt = Ct_target gene_ − Ct_GAPDH_. Ct refers to the amplification cycle when the real‐time fluorescence intensity reached the set threshold value and amplification entered a logarithmic growth phase.

**Table 3:** Primer sequences for GP73, TGF‐β1, Smad2, E‐cadherin, N‐cadherin, vimentin, WT1 and GAPDH genes

Gene	Forward	Reverse
GP73	GTGCTGGTGCCAGCCTGTTA	AGTGCTCTAGGCCATTGATTGATTG
TGF‐β1	AACCCACAACGAAATCTA	TGAGGTATCGCCAGGAAT
Smad2	CGTCCATCTTGCCATTCACG	CTCAAGCTCATCTAATCGTCCTG
E‐cadherin	AACGCATTGCCACATACAC	GAGCACCTTCCATGACAGAC
N‐ cadherin	TGCGCGTGAAGGTTTGCCAGT	TGGCGTTCTTTATCCCGGCGT
Vimentin	ACAGGCTTTAGCGAGTTATT	GGGCTCCTAGCGGTTTAG
WT1	CAAGGACTGCGAGAGAAGGTTT	TGGTGTGGGTCTTCAGATGGT
GAPDH	CCATGGAGAAGGCTGGGG	CAAAGTTGTCAT GGATGACC

GP73, Golgi membrane protein 73; TGF‐β1, transforming growth factor‐β1; GAPDH, glyceraldehyde‐3‐phosphate dehydrogenase; WT1, Wilms' tumour gene 1.

### Western blotting

T24, 5637, RT4, 253J and J82 cells were collected and total protein was collected after lysis and centrifugation. A BCA kit (Thermo Fisher Scientific, Sunnyvale, CA, USA) was used to detect the levels of GP73, E‐cadherin, N‐cadherin and vimentin for screening cell lines. The protein expression levels of GP73, TGF‐β1, Smad2, E‐cadherin, N‐cadherin, vimentin, p‐Smad2 and WT1 in transfected 5637 and 253J cells were determined.

Protein concentrations were measured using a bicinchoninic acid (BCA) kit (Thermo Fisher Scientific). Proteins were separated using 12% sodium dodecyl sulphate‐polyacrylamide gel electrophoresis (SDS‐PAGE), transferred to a polyvinylidene fluoride (PVDF) membrane and then blocked with skimmed milk at room temperature for 1 hr. The samples were incubated overnight with monoclonal antibodies for GAPDH (ab8245), GP73 (ab109628), TGF‐β1 (ab64715), Smad2 (ab76055), p‐Smad2 (ab53100), E‐cadherin (ab33875), vimentin (ab8978) and WT1 (ab201948) (1:300 dilution; Abcam Inc.) at 4°C. After washing three times, the samples were incubated with second antibodies at room temperature for 1 hr. After the membrane was washed for three times, an ECL kit was used to develop Western blotting bands. GAPDH was used as an internal control, and ImageJ2x software Rawak Software, Inc. Germany was used to calculate the relative expression levels of the target proteins.

### 3‐(4, 5‐Dimethylthiazol‐2‐yl)‐2, 5‐diphenyltetrazolium bromide (MTT) assay

The 253J and 5637 bladder cancer cells were seeded onto 96‐well plates at 48 hrs after transfection (5 × 10^3^/well). To each well, 200 μl of DMEM containing 10% FBS was added, and the cells were cultured at 37°C with 5% CO_2_ for 12, 24 and 48 hrs. Five wells were set for each concentration. Each well was supplemented with 20 μl of MTT (PBS diluted to 5 g/l) in the dark, and the plate was incubated for 4 hrs. After discarding the supernatant, 150 μl of dimethyl sulfoxide (DMSO) was added to each well, and samples were allowed to dissolve for 10 min. The OD at 490 nm was measured using an enzyme‐linked immunometric metre. A cell survival curve was constructed, with time‐points as the abscissa and OD values as the ordinate.

### Scratch test

253J and 5637 cells were collected at 48 hrs after transfection, and cell suspensions were seeded onto 6‐well plates at a density of 1 × 10^5^ cells/well. When the cells reached 90% confluency, a 200‐μl pipette tip was used to scratch four horizontal and four vertical streaks on the well surface. The widths at multiple scratch points were measured, and the scratch healing rates [(scratch width at 0 hrs—scratch width at 24 hrs)/scratch width at 0 hr × 100%] were calculated. The mean values for three duplicate assays were conducted to compare cell migration ability in each group.

### Matrigel invasion assay

Matrigel was dissolved overnight at 4°C and then diluted with serum‐free DMEM (1:3). The diluted Matrigel was added to the upper chamber of the Transwell to form the membrane in three additions (15, 7.5 and 7.5 μl), with 10 min. between each addition. The Matrigel was evenly spread and covered all micropores at the bottom of the upper chamber. 253J and 5637 cells were collected at 48 hrs after transfection to prepare cell suspensions. Cell suspensions were seeded into the upper well, and 0.5 ml of DMEM containing 10% FBS was added to the lower chambers (24‐well plates). The cells were cultured routinely for 48 hrs at 37°C with 5% CO_2_. After using a cotton swab to gently wipe off the remaining free cells in the upper well, the well membrane was collected and fixed for 15 ~ 20 min. in 95% ethanol. After rinsing with clean water, the membrane was stained for 10 min. and washed again with clean water. The membrane was then observed under a high‐powered inverted microscope to count the number of cells on the membrane. The invasion ability of the cells was determined by counting the number of cells that passed through the Matrigel, and the average number from five high‐magnification fields of view was used for each sample.

### Statistical analysis

All data were analysed using SPSS 21.0 statistical software (SPSS Inc., Chicago, IL, USA). Data with a normal distribution were analysed using a K‐S test. Data are presented as mean values with standard deviations (S.D.). Comparisons among multiple groups were performed using a one‐way analysis of variance (ANOVA), and comparisons between two groups were performed using *t*‐tests. The categorical data were compared using a chi‐square test. A value of *P* < 0.05 was considered to be statistically significant.

## Results

### High expressions of GP73, TGF‐β1, Smad2 and vimentin, and low expression of E‐cadherin are found in bladder cancer tissues

**Figure 1:**
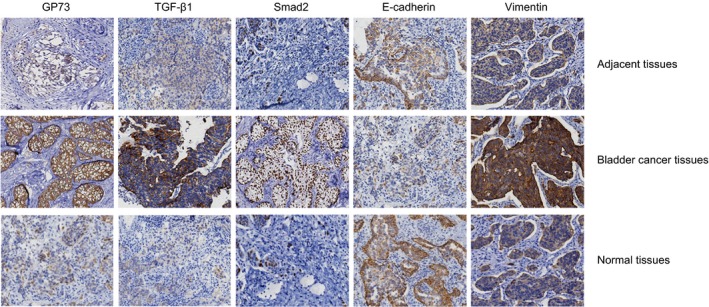
Protein levels of GP73, TGF‐β1, Smad2, E‐cadherin and vimentin in bladder cancer tissues and in adjacent and normal bladder tissues detected by IHC assays (× 400). Note: GP73, Golgi membrane protein 73; TGF‐β1, transforming growth factor‐β1; IHC, immunohistochemistry.

Positive staining for GP73, TGF‐β1 and Smad2 was observed in the Golgi apparatus, cytoplasm and nucleus, respectively (Fig. 1). The expression levels of GP73, TGF‐β1 and Smad2 in bladder cancer tissue samples were significantly higher than those in adjacent and normal bladder tissue samples (61.8% *versus* 10.8% *versus* 3.8%, 72.5% *versus* 19.6% *versus* 7.5% and 65.7% *versus* 15.7% *versus* 6.6%, respectively) (all *P* < 0.05) (Table 4). Positive staining for E‐cadherin was observed in cell membranes, and the expression level of E‐cadherin in bladder cancer tissue samples (18.6%, 19/102) was significantly lower than that in adjacent (53.9%, 55/102) and normal bladder tissue samples (76.4%, 81/106) (*P* < 0.001). Positive staining of vimentin was observed in the cytoplasm, and vimentin expression in bladder cancer tissue samples (71.6%, 73/102) was significantly higher than that in adjacent (21.6%, 22/102) and normal bladder tissue samples (11.3%, 12/106) (*P* < 0.05). Sarcomas were observed in highly differentiated and moderately differentiated bladder cancer tissue samples.

**Table 4:** The positive expression rates of GP73, TGF‐β1, Smad2, E‐cadherin and vimentin proteins in bladder cancer tissues, adjacent tissues and normal bladder tissues

Protein	Bladder cancer tissues	Adjacent tissues	Normal bladder tissues	χ^2^ value	*P* value
GP73					
+	63	11	4	109.5	<0.001
−	39	91	102		
TGF‐β1					
+	70	20	8	99.84	<0.001
−	32	82	98		
Smad2					
+	66	16	7	99.83	<0.001
−	36	86	99		
E‐cadherin					
+	19	39	81	70.37	<0.001
−	83	63	25		
Vimentin					
+	73	22	12	94.75	<0.001
−	29	80	94		

GP73, Golgi membrane protein 73; TGF‐β1, transforming growth factor‐β1.

### Expressions of GP73, TGF‐β1 and Smad2 correlate with bladder cancer development

The expression levels of GP73, TGF‐β1 and Smad2 in HG urothelial carcinomas were significantly higher than those in LMP papillary urothelial cancers and LG urothelial carcinomas (all *P* < 0.05). The expression levels of GP73, TGF‐β1 and Smad2 in MIBC patients were higher than those in NMIBC patients (all *P* < 0.05). The expression levels of GP73, TGF‐β1 and Smad2 in stage III‐IV cases were higher than those in stage I‐II cases (all *P* < 0.05). The expression levels of GP73, TGF‐β1 and Smad2 in patients with LNMs were also significantly higher than those in patients without LNMs (all *P* < 0.05). The expression levels of GP73, TGF‐β1 and Smad2 were not associated with gender or age (all *P* > 0.05) (Table 5).

**Table 5:** Correlations between the protein expressions of GP73, TGF‐β1 and Smad2 with clinicopathological characteristics of patients with bladder cancer

Characteristic	n	GP73		Positive rates (%)	*P*	TGF‐β1		Positive rates (%)	*P*	Smad2		Positive rates (%)	*P*
		+	−			+	−			+	−		
Age (year)													
<65	49	30	19	61.22	0.914	34	15	69.39	0.491	30	19	61.22	0.361
≥ 65	53	33	20	62.26		40	13	75.47		37	16	69.81	
Gender													
Male	54	34	20	62.96	0.792	38	16	70.37	0.601	34	20	62.96	0.539
Female	48	29	19	60.42		36	12	75.00		33	15	68.75	
Pathologic stage													
LMP	17	5	12	29.41	<0.001	9	8	52.94	0.009	8	9	47.06	0.010
LG	35	19	16	54.29		22	13	62.86		19	16	54.29	
HG	50	39	11	78.00		43	7	86.00		40	10	80.00	
MIBC													
Yes	52	26	26	50.00	0.013	31	21	59.62	0.003	28	24	53.85	0.010
No	50	37	13	74.00		43	7	86.00		39	11	78.00	
TNM stage													
I–II stage	65	28	37	43.08	0.013	42	23	64.62	0.003	38	27	58.46	0.010
III–IV stage	37	35	2	94.59		32	5	86.49		29	8	78.38	
LNM													
Yes	35	32	3	91.43	<0.001	31	4	88.57	0.009	28	7	80.00	0.028
No	67	31	36	46.27		43	24	64.18		39	28	58.21	

GP73, Golgi membrane protein 73; TGF‐β1, transforming growth factor‐β1; LMP, low malignant potential; LG, low grade; HG, high grade; MIBC, muscle‐invasive bladder cancer; TNM, tumour node metastasis; LNM, lymph node metastasis.

### 5637 and 253J cells showing expressions of GP73, E‐cadherin, N‐cadherin and vimentin are selected for *in vitro* experiments

**Figure 2:**
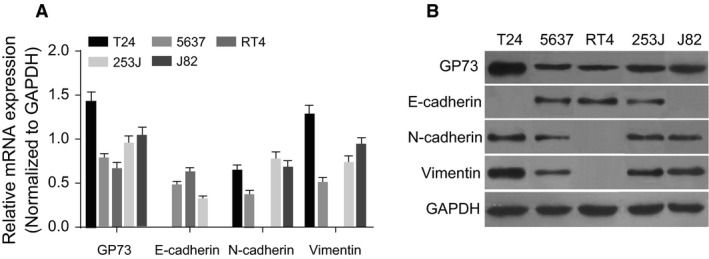
Expression levels of GP73, E‐cadherin, N‐cadherin and vimentin in T24, 5637, RT4, 253J and J82 cells. Note: (**A**) mRNA expression for GP73, E‐cadherin, N‐cadherin and vimentin was detected by qRT‐pPCR; (**B**) protein expression of GP73, E‐cadherin, N‐cadherin and vimentin was detected by Western blotting; GP73, Golgi membrane protein 73; RT‐qPCR, reverse transcription quantitative polymerase chain reaction.

RT‐qPCR and Western blotting were used to detect the mRNA and protein levels of GP73, E‐cadherin, N‐cadherin and vimentin in the 5637, 253J, RT4, J82 and T24 cell lines (Fig. 2). The results indicate that the above five cell types expressed GP73, while no expression level of E‐cadherin was detected in T24 and J82 cells. In addition, RT4 cells exhibited no expression level of vimentin or N‐cadherin. Therefore, 5637 and 253J cells, which exhibited GP73, E‐cadherin, N‐cadherin and vimentin, were selected for further experiments.

### WT1 siRNA‐3 and 48‐h transfection are prepared for *in vitro* experiments

**Figure 3:**
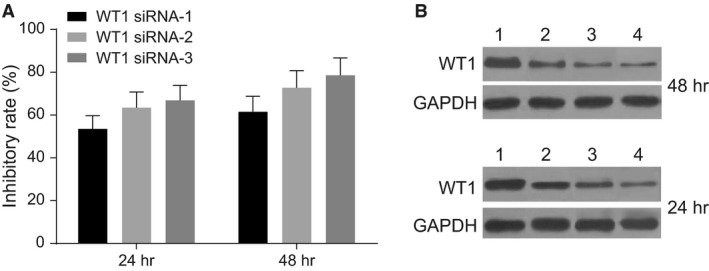
Protein levels of WT1 24 and 48 hrs after transfection of siRNA‐1, ‐2 and ‐3 against WT1, determined using Western blotting. Note: **A**, the protein levels of WT1; **B**, grey values of WT1 protein bands; 1, NC siRNA; 2, siRNA‐1 against WT1; 3, siRNA‐2 against WT1; 4, siRNA‐3 against WT1; WT1, Wilms' tumour gene 1; NC, negative control.

Twenty‐four and 48 hours after transfection, the protein levels of WT1C, determined using Western blotting, are shown in the Figure 3. Twenty‐four hours after transfection, we found that the inhibitory rates of siRNA‐1, ‐2 and ‐3 against WT1 reached 53.51%, 63.43% and 66.86%, respectively. Forty‐eight hours after transfection, the inhibitory rates of siRNA‐1, ‐2 and ‐3 against WT1 reached 61.55%, 72.77% and 78.63%, respectively. Therefore, WT1 siRNA‐3 and 48‐h transfection were selected for further experiments.

### The results of RT‐qPCR: GP73 modulates EMT in 5637 and 253J cells by down‐regulating WT1 and up‐regulating TGF‐β1 and Smad2

**Figure 4:**
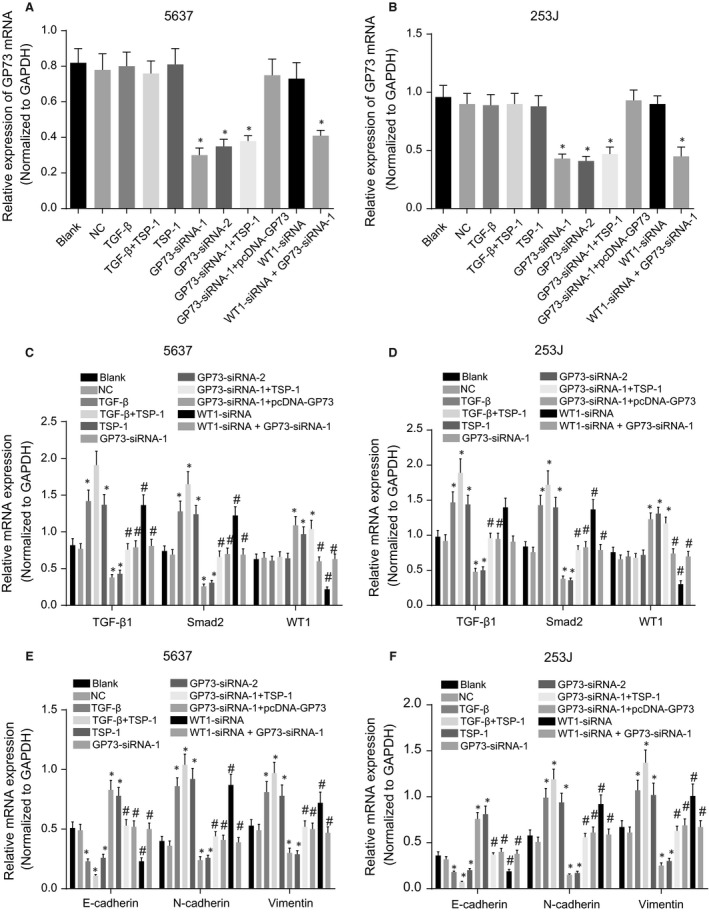
mRNA levels of GP73, TGF‐β1, Smad2, E‐cadherin, N‐cadherin and vimentin in 5637 and 253J cells 48 hrs after transfection. Note: (**A**) GP73 mRNA level in 5637 cells; (**B**) GP73 mRNA level in 253J cells; (**C**), mRNA levels of TGF‐β1, Smad2 and WT1 in 5637 cells; (**D**) mRNA levels of TGF‐β1, Smad2 and WT1 in 253J cells; (**E**) mRNA levels of vimentin, N‐cadherin and E‐cadherin in 5637 cells; (**F**), mRNA levels of vimentin, N‐cadherin and E‐cadherin in 253J cells; *, *P* < 0.05, compared with the blank and NC groups; ^#^, *P* < 0.05, compared with the GP73‐siRNA‐1 group; NC, negative control; TSP‐1, thrombospondin‐1; GP73, Golgi membrane protein 73; WT1, Wilms' tumour gene 1; TGF‐β1, transforming growth factor‐β1.

RT‐qPCR analysis demonstrated that in 5637 and 253J cells 48 hrs after transfection, compared to expression in the blank and NC groups, the mRNA level of GP73 significantly decreased, but WT1 mRNA level increased in the GP73‐siRNA‐1, GP73‐siRNA‐2 and GP73‐siRNA‐1+ TSP‐1 groups; WT1 mRNA level decreased in the WT1‐siRNA group; GP73 mRNA level decreased in the WT1‐siRNA + GP73‐siRNA‐1 group (all *P* < 0.05); WT1 mRNA level showed no difference in the WT1‐siRNA + GP73‐siRNA‐1 group (*P* > 0.05); and there were no significant differences in GP73 and WT1 mRNA level among the other groups (all *P* > 0.05). Compared with the blank and NC groups, mRNA levels of TGF‐β1 and Smad2, vimentin and N‐cadherin increased and E‐cadherin mRNA level decreased in the WT1‐siRNA, TSP‐1, TGF‐β and TGF‐β + TSP‐1 groups, and the TGF‐β + TSP‐1 group showed the most significant changes (all *P* < 0.05). Compared with the blank and NC groups, mRNA level of TGF‐β1, Smad2, vimentin and N‐cadherin decreased remarkably, and expression level of E‐cadherin increased in the GP73‐siRNA‐1 and GP73‐siRNA‐2 groups. In comparison with the GP73‐siRNA‐1 group, the WT1‐siRNA, GP73‐siRNA‐1+ TSP‐1 and GP73‐siRNA‐1+ pcDNA‐GP73 groups showed up‐regulated mRNA levels of TGF‐β1, Smad2, vimentin and N‐cadherin and down‐regulated mRNA level of E‐cadherin, and these changes were more remarkable in the WT1‐siRNA group (all *P* < 0.05). mRNA levels of TGF‐β1, Smad2, vimentin, N‐cadherin and E‐cadherin in the GP73‐siRNA‐1+ pcDNA‐GP73 and GP73‐siRNA‐1+ TSP‐1 groups showed no significant difference in comparison with the NC group (all *P* < 0.05) (Fig. 4).

### The results of Western blotting: GP73 modulates EMT in 5637 and 253J cells by down‐regulating WT1 and up‐regulating TGF‐β1 and Smad2

**Figure 5:**
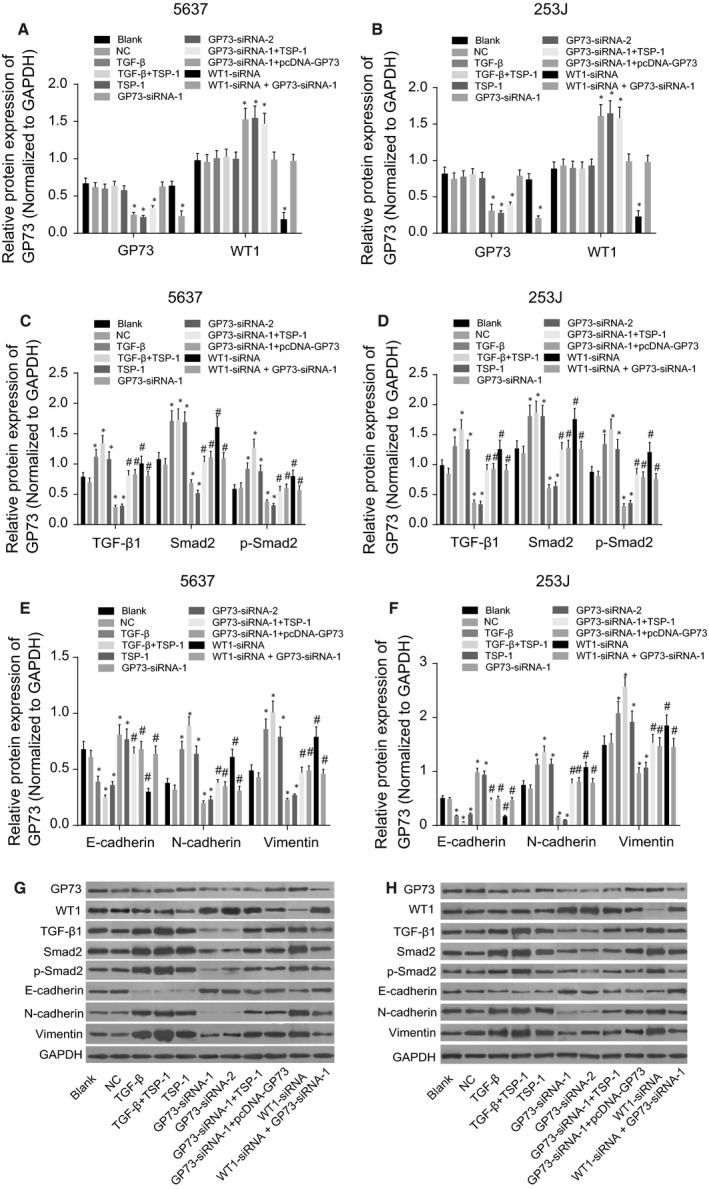
Protein levels of GP73, WT1, TGF‐β1, Smad2, p‐Smad2, E‐cadherin, N‐cadherin and vimentin in 5637 and 253J cells 48 hrs after transfection. Note: (**A**) protein levels of GP73 and WT1 in 5637 cells; (**B**) protein levels of GP73 and WT1 in 253J cells; (**C**) protein levels of TGF‐β1, Smad2 and p‐Smad2 in 5637 cells; (**D**) protein levels of TGF‐β1, Smad2 and p‐Smad2 in 253J cells; (**E**) protein levels of vimentin, N‐cadherin and E‐cadherin in 5637 cells; (**F**), protein levels of vimentin, N‐cadherin and E‐cadherin in 253J cells; (**G**), protein band pattern of 5637 cells; (**H**), protein band pattern of 253J cells; *, *P* < 0.05, compared with the blank and NC groups; ^#^, *P* < 0.05, compared with the GP73‐siRNA‐1 group; NC, negative control; TSP‐1, thrombospondin‐1; GP73, Golgi membrane protein 73; WT1, Wilms' tumour gene 1; TGF‐β1, transforming growth factor‐β1.

Western blotting results demonstrated that 48 hrs after transfection in both 5637 and 253J cells, compared with the blank and NC groups, the GP73‐siRNA‐1, GP73‐siRNA‐2 and GP73‐siRNA + TSP‐1 groups had decreased GP73 protein level and increased WT1 protein level; WT1 protein level was decreased in the WT1‐siRNA group; GP73 protein level decreased in the WT1‐siRNA + GP73‐siRNA‐1 group (all *P* < 0.05); WT1 protein level showed no difference in the WT1‐siRNA + GP73‐siRNA‐1 group (*P* > 0.05), and there were no significant differences in GP73 protein level among the other groups (all *P* > 0.05). Compared with the blank and NC groups, the protein level of TGF‐β1, Smad2, p‐Smad2, vimentin and N‐cadherin increased and that of E‐cadherin decreased in the WT1‐siRNA, TSP‐1, TGF‐β and TGF‐β + TSP‐1 groups, and these changes were the most significant in TGF‐β + TSP‐1 group (all *P* < 0.05). The GP73‐siRNA‐1 and GP73‐siRNA‐2 groups showed reduced protein level of TGF‐β1, Smad2, p‐Smad2, vimentin and N‐cadherin and increased E‐cadherin protein level (all *P* < 0.05). In comparison with the GP73‐siRNA‐1 group, the WT1‐siRNA, GP73‐siRNA + TSP‐1 and GP73‐siRNA‐1+ pcDNA‐GP73 groups showed up‐regulated protein level of TGF‐β1, Smad2, p‐Smad2, vimentin and N‐cadherin but down‐regulated E‐cadherin protein level, and the WT1‐siRNA group exhibited the most significant changes (all *P* < 0.05). No significant differences were detected among the blank, WT1‐siRNA + GP73‐siRNA‐1, GP73‐siRNA‐1+ pcDNA‐GP73 and GP73‐siRNA‐1+ TSP‐1 groups for the protein level of TGF‐β1, Smad2, vimentin, N‐cadherin and E‐cadherin (all *P* > 0.05) (Fig. 5). Based on these results, it can be concluded that silencing GP73 inhibits protein levels of TGF‐β1, Smad2, p‐Smad2 in 5637 and 253J cells, leading to the EMT. The addition of the activator TSP‐1 can induce the EMT in 5637 and 253J cells.

### GP73 promotes cell proliferation in 5637 and 253J cells

**Figure 6:**
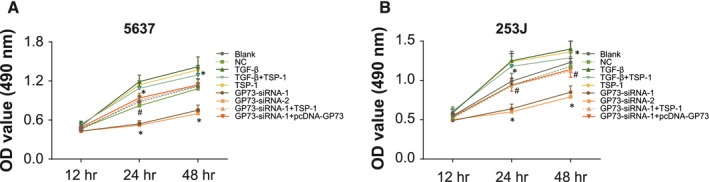
Proliferation of 5637 and 253J cells determined by MTT assay 48 hrs after transfection. Note: (**A**) cell growth curve for 5637 cells; (**B**) cell growth curve for 253J cells; *, *P* < 0.05 compared with the blank and negative control groups; ^#^, *P* < 0.05 compared with the GP73‐siRNA‐1 group; NC, negative control; TSP‐1, thrombospondin‐1; GP73, Golgi membrane protein 73; MTT, 3‐(4,5‐dimethylthiazol‐2‐yl)‐2,5‐diphenyltetrazolium bromide; TGF‐β, transforming growth factor‐β; OD, optical density.

MTT assay results indicated that at 24 and 48 hrs after transfection, the proliferation of 5637 and 253J cells in the TSP‐1, TGF‐β and TGF‐β + TSP‐1 groups was enhanced; however, the speed of proliferation started to slow down after 24 hrs; changes in the TGF‐β and TGF‐β + TSP‐1 group were the most remarkable. In the GP73‐siRNA‐1 and GP73‐siRNA‐2 groups, proliferation was inhibited compared with the blank and NC groups, and differences in OD value were statistically significant at 24 and 48 hrs (all *P* < 0.05). Compared with the GP73‐siRNA‐1 group, proliferation in the GP73‐siRNA‐1+ TSP‐1 and GP73‐siRNA‐1+ pcDNA‐GP73 groups increased (all *P* < 0.05). Silencing GP73 inhibits the proliferation of 5637 and 253J cells. In addition, 253J cell proliferation significantly increased initially with TSP‐1 stimulation, but it began to decrease after the EMT (Fig. 6).

### GP73 promotes cell migration in 5637 and 253J cells

**Figure 7:**
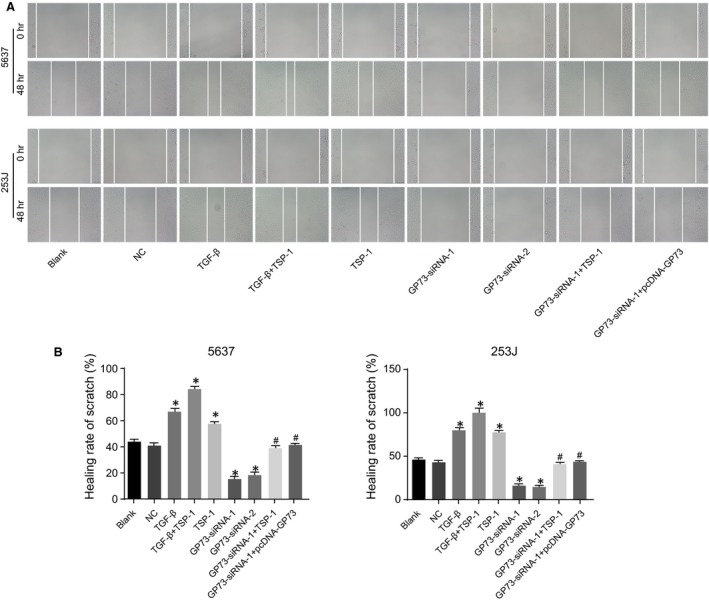
Migration of 5637 and 253J cells detected by scratch tests 48 hrs after transfection (× 40). Note: (**A**) scratch test images of 5637 and 253J cells at 48 hrs after transfection; (**B**) statistical results for scratch healing rates of 5637 and 253J cells 48 hrs after transfection; *, *P* < 0.05 compared with the blank and NC groups; ^#^, *P* < 0.05 compared with the GP73‐siRNA‐1 group; NC, negative control; TSP‐1, thrombospondin‐1; GP73, Golgi membrane protein 73; TGF‐β, transforming growth factor‐β.

As shown in the scratch test results in Figure 7, at 24 hrs after incubation, compared with the blank and NC groups, the healing rates of 5637 and 253J cells were higher in the TSP‐1, TGF‐β and TGF‐β + TSP‐1 groups but lower in the GP73‐siRNA‐1 and GP73‐siRNA‐2 groups, and rates significantly increased in the TGF‐β + TSP‐1 group. Compared with the GP73‐siRNA‐1 group, the GP73‐siRNA‐1+ TSP‐1 and GP73‐siRNA‐1+ pcDNA‐GP73 groups showed increased healing rates (all *P* < 0.05). This suggests that silencing GP73 inhibits the migration ability of 5637 and 253J cells, while TSP‐1 significantly increases the migration ability of 5637 and 253J cells.

### GP73 promotes cell invasion in 5637 and 253J cells

**Figure 8:**
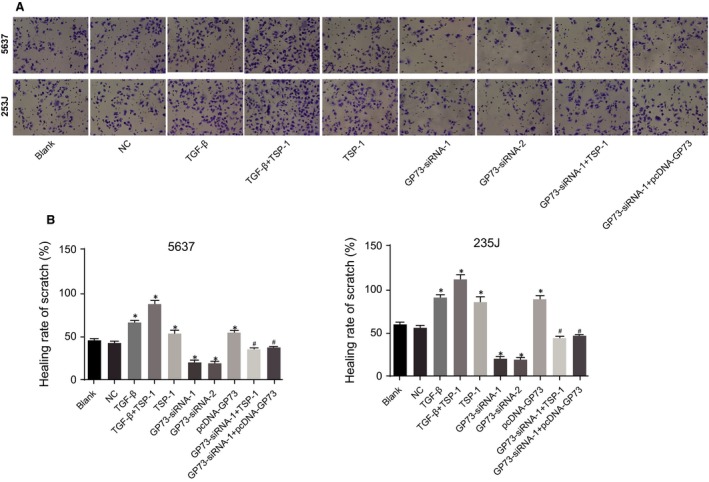
Invasion of 5637 and 253J cells detected by Matrigel assays 48 hrs after transfection (× 200). Note: (**A**) invasion images of 5637 and 253J cells 48 hrs after transfection; (**B**) statistical results for the number of invasive 5637 and 253J cells 48 hrs after transfection; *, *P* < 0.05, compared with the blank and NC groups; ^#^, *P* < 0.05, compared with the GP73‐siRNA‐1 group; NC, negative control; TSP‐1, thrombospondin‐1; GP73, Golgi membrane protein 73; TGF‐β, transforming growth factor‐β.

As shown in Figure 8, at 48 hrs after incubation in the Matrigel assay, compared with the blank and NC groups, more cells penetrated the membrane in the TSP‐1, TGF‐β and TGF‐β + TSP‐1 groups, but fewer cells crossed in the GP73‐siRNA‐1 and GP73‐siRNA‐2 groups, and the increase in invasion ability was most notable in the TGF‐β + TSP‐1 group (all *P* < 0.05). Compared with the GP73‐siRNA‐1 group, the GP73‐siRNA‐1+ TSP‐1 and GP73‐siRNA‐1+ pcDNA‐GP73 groups had more cells penetrate through the membrane (all *P* < 0.05), which suggested that silencing GP73 inhibits the invasion ability of 5637 and 253J cells, and TSP‐1 promotes the invasion ability of these cells.

## Discussion

Bladder cancer has the characteristics of invasive growth and rapid recurrence and tends to occur simultaneously in multiple locations 28. Due to its complex mechanism of progression, the specific molecular mechanisms of its pathogenesis are not fully understood. As there are no molecularly targeted agents approved for treating bladder cancer, The Cancer Genome Atlas (TCGA) project was conducted to identify potential therapeutic targets for this disease 29. There is evidence supporting the fact that developments in molecular biology, genomics, bioinformatics and immunology may provide a solid foundation for therapeutic advances in treating this disease 30. Therefore, the aim of this study was to investigate how GP73/TGF‐β1/Smad2 regulates the EMT and promotes the invasion and metastasis of bladder cancer. It is hoped that such research may provide a theoretical foundation for the search for new molecular and therapeutic targets for bladder cancer.

In this study, the associations between GP73/TGF‐β1/Smad2 and the clinic pathological features of patients with bladder cancer were explored. It was determined that the above three factors were closely related to the clinical stage, pathological stage and presence of LNMs in bladder cancer. It has been previously reported that TGF‐β1, Smad2 and GP73 were significantly correlated with the clinical staging, grading and LNMs of tumours, which is consistent with the results from this study 15, 31, 32.

This study also demonstrated that GP73 was highly expressed in bladder cancer tissues. GP73 is a resident Golgi‐specific membrane protein that is generally expressed in normal epithelial cells 33. However, it has been shown that GP73 is also highly expressed in HCC and malignant tissues 19. There is evidence that the expression of GP73 increases at different stages and can be an accurate serum marker for the detection of HCC and its recurrence after surgery 34. The up‐regulation of GP73 in cancer cells may be related to the stability and integrity of the Golgi complex, and changes in gene expression levels and micro environments may reflect the metabolic requirements of the cancer cells 35, 36. This study demonstrated that TGF‐β1 and Smad2 were highly expressed in bladder cancer tissues, and RT‐qPCR and Western blotting demonstrated that the mRNA and protein levels of TGF‐β1, Smad2 and p‐Smad2 increased in the TSP‐1, TGF‐β and TGF‐β + TSP‐1 groups, while they decreased in the GP73‐siRNA‐1 and GP73‐siRNA‐2 groups. It has been reported that TSP‐1 enhances the EMT in human mesothelial cells and can promotes an aggressive phenotype through the EMT in human melanoma 37, 38. GP73 is encoded by the GOLPH2 gene. Previous study has demonstrated that GOLPH2 is related to WT1, and knockdown of GOLPH2 can up‐regulate WT1 expression 20. The CGCCCCCGC response element in the WT1 protein inhibits the expression of TGF‐β1 by spanning nucleotides ‐111 to ‐119 of the TGF‐β1 promoter 39. Studies have shown that TGF‐β1 is expressed in all tumour cells and functions at all tumours stages, showing an inhibitory effect in early tumour cell proliferation and inducing apoptosis, whereas it promotes the proliferation, invasion and metastasis of tumour cells in later stages 40, 41. It was found that high TGF‐β1 expression levels promoted the growth of tumour cells by affecting molecules in the TGF‐β1 signalling pathway during the occurrence and progression of tumours, resulting in the abnormal proliferation of tumour cells; however, the proliferation of tumour cells also promoted TGF‐β1 secretion and accelerated the invasion of tumour cells into normal tissues 42. The activation of TGF‐β signalling is considered a critical event during the EMT 43, and TGF‐β1 acts as an inducer of the EMT in several tissues 44, 45. The Smad signalling pathway is the main mechanism underlying the TGF‐β1‐induced initiation of the EMT, in which TGF‐β1 induces the activation of its receptor, activates the phosphorylation of Smad proteins through receptor regulation, affects the transcription of genes in the nucleus and eventually contributes to the TGF‐β1‐induced EMT 46. Similarly, Smad2 was found to play an important role in TGF‐β1/Smad signal transmission, and its mutation or loss of expression could disrupt the relay of signals and reduce chemokine levels in the cells 47, 48. In this study, Smad2 was highly expressed in bladder cancer tissue, which may be due to abnormal Smad2 signalling function during carcinogenesis, thus decreasing the inhibitory effects of the TGF‐β1/Smad pathway on cancer cells, enhancing the feedback signals from TGF‐β1 and increasing the expression of Smad2. Lv *et al*. found that high levels of TGF‐β1 in cancer cells may lead to a poor prognosis. By treating bladder cancer cells with TGF‐β1, they found that mesenchymal staining increased while the markers for epithelial cells decreased, indicating that the morphology of the cells changed and their invasiveness increased 49. However, by inhibiting the expression of Smad2, they were able to prevent such events from occurring 49.

The effect of GP73/TGF‐β1/Smad2 signalling on the expression of EMT biomarkers was explored. The TSP‐1, TGF‐β and TGF‐β + TSP‐1 groups had decreased E‐cadherin expression level but increased expression levels of vimentin and N‐cadherin, while the GP73‐siRNA‐1 and GP73‐siRNA‐2 groups had a trend completely opposite that of these three groups, further confirming the role of GP73/TGF‐β1/Smad2 signalling. The EMT is a complex process that is accompanied by the loss of epithelial markers, such as the adherent junction protein E‐cadherin, and the acquisition of mesenchymal markers, such as vimentin and fibronectin 50. Reduced expression of E‐cadherin leads to an adenoma‐to‐carcinoma transition in animals 51. The transition from E‐cadherin to N‐cadherin is involved in the transformation of normal, non‐progressive cells to a malignant and invasive cancer cells 52. The expression of vimentin, an archetypal mesenchymal marker, is preceded by the loss of epithelial features and contributes to the overexpression of mesenchymal genes 53. Our results imply that silencing GP73 can significantly inhibit cell growth, cell migration and cell invasion of human bladder cancer 5637 and 253J cells, but that stimulation of TSP‐1 can enhance cell growth, migration and invasion. At present, numerous studies have shown that TSP‐1 is an important factor in the activation of TGF‐β1 through both *in vitro* cell cultures and in an inflammatory nephritis model 54, 55, 56, 57. It is known that the EMT is a pivotal mechanism that contributes to cancer invasion and metastasis 19. Bao *et al*. 19 demonstrated that the expression of GP73 in liver cancer cells was positively correlated with the expression of vimentin and EMT‐related molecules and was negatively correlated with E‐cadherin expression, indicating that a high level of GP73 expression in cancer tissues may be related to the occurrence, development, invasion and metastasis of cancer. In addition, Liu *et al*. 58 demonstrated that the expression of GP73 increased the expression of EMT‐related factors and that GP73 silencing contributed to the inhibition of tumour proliferation and metastasis. It has been suggested that the interaction of GP73 with other pivotal proteins directly in the EMT can play a role in this process as GP73, as a Golgi transmembrane protein, is unlikely to be directly involved in EMT‐inducing signalling pathways 59. Furthermore, TGF‐β1 has also been reported to initiate and maintain the EMT in a variety of biological systems and pathophysiological contexts by activating major signalling pathways 60. Normally, TGF‐β exerts anticancer activities by inhibiting cell proliferation, cell motility, invasion and metastasis; however, growing evidence indicates that the process of tumorigenesis or metastatic progression allows TGF‐β to exhibit oncogenic activities 61. Previous studies have demonstrated that GP73 expression correlates with the gene expression of TGF‐b1, and the expression of GP73 seems to be able to respond to some cytokines 62, 63. Increasing TGF‐b levels may enhance the expression of GP73, and given that the TGF‐b can promote cell invasion, the researchers speculated that cancer cells can stimulate GP73 secretion in some way 31, 64, 65. Our results are consistent with these findings.

In summary, our results provide evidence that GP73 can activate the TGF‐β1/Smad2 signalling pathway and induce the EMT *via* down‐regulating WT1 expression, thereby promoting the invasion and metastasis of bladder cancer. Furthermore, GP73, TGF‐β1 and Smad2 were highly expressed in bladder cancer and were associated with clinical features such as the clinical and pathological stages and LNMs of bladder cancer. This study may provide a theoretical basis for a better in‐depth understanding of the molecular mechanisms that are involved in bladder cancer and for finding new molecular targets for clinical applications. However, this study did not explore other potential signalling pathways that might be involved in this process, and further studies are required to understand these detailed mechanisms. Furthermore, the number of cases included in this study and the fact that only two cell lines were used for the mechanistic experiments may affect the reliability of our findings. Therefore, our results must be further verified and enriched with findings from studies with larger sample sizes to enhance the reliability of our results.

## Competing interests

None.
